# Metabolic syndrome patterns by gender in major depressive disorder

**DOI:** 10.1371/journal.pone.0313629

**Published:** 2024-12-04

**Authors:** Lu Li, Ling Xiong, Zhihua Liu, Lin Zhang

**Affiliations:** 1 Department of Psychiatry, Wuhan Mental Health Center, Wuhan, China; 2 Department of Anesthesiology, The Affiliated Hospital of Hubei University of Chinese Medicine, Wuhan, China; 3 Department of Psychiatry, The Fourth People’s Hospital of Nanyang, Nanyang, Henan, China; Tribhuvan University Institute of Medicine, NEPAL

## Abstract

Major depressive disorder (MDD) and metabolic syndrome (MetS) are significant health challenges, with distinct gender-specific manifestations. This suggests that the clinical presentation of MetS within the MDD cohort may also vary by gender. The objective of this study is to explore these gender-specific clinical patterns in the co-occurrence of MetS among hospitalized MDD patients, thereby offering insights and guidance for targeted interventions aimed at managing MetS in this demographic. The study included 1,281 first hospitalization MDD patients. Data were collected on socio-demographic characteristics and general clinical profiles. Metabolic parameters, routine biochemical markers, and psychological symptoms were measured and analyzed. The prevalence of MetS was 8.21% in male patients and 10.34% in female patients, with no significant difference between genders. Gender-specific risk factors were identified: in males, age and anxiety symptoms were significant predictors of MetS, while in females, age at onset and married were linked to the development of MetS. Additionally, MetS severity was influenced by age at onset in males and by both age at onset and married in females. This study found no gender-specific prevalence of MetS in hospitalized MDD patients. However, gender-specific factors influencing MetS development and severity highlight the need for focused management in older, married females and older males with high anxiety symptoms.

## 1. Introduction

Major depressive disorder (MDD) is a severely disabling psychiatric condition [1], affecting around 6% of the global population and contributing significantly to disability-adjusted life years among mental health disorders [2, 3]. Individuals with MDD face a reduced life expectancy of 10–14 years compared to the general population [4]. Despite extensive research, the pathophysiological mechanisms of MDD remain largely unclear, with the disorder characterized by a high recurrence rate and only about one-third of patients achieving remission following treatment [5, 6]. MDD patients also have the highest risk of all-cause mortality and suicide among psychiatric conditions [7].

Metabolic syndrome (MetS) is a cluster of risk factors for cardiovascular disease (CVD) and diabetes mellitus [8–10]. and as healthcare systems advance globally, MetS has become a significant modern health concern [11]. Approximately one-fourth of the global population is affected by MetS, with its prevalence steadily increasing [12–14]. In China, a national survey showed a rise in MetS prevalence from 13.7% in 2000–2001 to 31.1% in 2015–2017 [15, 16], highlighting the urgent need for targeted interventions.

Indeed, a substantial body of research has been conducted on the topic of MetS in individuals with MDD. MetS and MDD are thought to share a common genetic pathway [17–19], and both are causative of each other, contributing to each other’s deterioration and progression, while MetS increases CVD morbidity and mortality in people with MDD [20–22]. In addition, researchers have identified several effects of MetS or its components on MDD. For instance, MDD is strongly associated with type 2 diabetes [23]. Indicators such as fasting blood glucose (FBG) levels, high-density lipoprotein cholesterol (HDL-c) levels and low plasma total cholesterol (TC) levels have been used to predict the risk of suicide in MDD patients [24–26]. These findings emphasize the necessity for further investigation into the contribution of metabolic disorders to the etiology of MDD. Accordingly, we hypothesize that effective management of the MetS in patients with MDD is a crucial initiative to reduce the incidence of cardiovascular disease, improve prognosis, and prevent unexpected events.

Gender differences are a well-recognized characteristic of MDD and MetS, attracting significant attention from psychiatrists and sociologists in their research endeavors [27, 28]. Females, compared to males, exhibit a higher prevalence of MDD [29], along with an increased risk of comorbid anxiety [30], suicide attempts [31], and poorer social functioning [32], among other things [33]. Similarly, gender-related differences were observed in MetS, as had been seen in MDD [15, 16, 34, 35]. Drawing from existing research on gender differences in MDD and MetS and inspired by prior reports on the clinical features of MetS in MDD patients [36], we had a keen interest in exploring gender-related differences in the clinical patterns of MetS in the MDD population. The knowledge obtained from our study may prove useful in the development of gender-specific therapeutic strategies for MetS in this particular demographic.

## 2. Participants and methods

### 2.1 Subjects

We based the sample size prediction on the following formula:

$$n=\frac{Z^{2}P(1-P)}{d^{2}}$$

where: n = Sample size, Z = Z-statistic for the chosen level of confidence (1.96 for a 95% confidence level), P = Expected prevalence or proportion (With reference to the prevalence of MetS in the Chinese general population, the value of 0.3 was taken here.), d = Precision or margin of error (equal to 0.05).Thus, the estimated sample size required was 323 participants.

This study included 1,281 admissions to the Wuhan Mental Health Center (a 950-bed tertiary psychiatric specialty hospital in central China, located in Wuhan, Hubei Province) and the Nanyang No. 4 People’s Hospital (a 660-bed tertiary psychiatric specialty hospital located in Nanyang City, Henan Province) from 06/07/2017, to 06/08/2023, all of whom were diagnosed as MDD patients.

To qualify for participation in the study, individuals had to satisfy the following conditions:

1. meet the diagnostic criteria for MDD as outlined in the 10th edition of the International Classification of Diseases (ICD-10).
2. no previous history of hospitalization.
3. be aged between 18 and 60 years and of Chinese Han ethnicity.
4. have a total score of 24 or higher on the 17-item Hamilton Depression Rating Scale (HAMD-17).

The study excluded any patients with the following characteristics:

1. women who were breastfeeding or pregnant.
2. individuals with a history of substance abuse.
3. those with significant physical illnesses or personality disorders.
4. patients previously diagnosed with diabetes mellitus.
5. individuals unable to cooperate with psychological evaluations due to severe behavioral issues or other factors.

The Ethics Committee of The Fourth People’s Hospital of Nanyang approved this research, and all participants provided written informed consent. Participants retained the right to withdraw from the study at any time.

### 2.2 Research design

**Fig 1:**
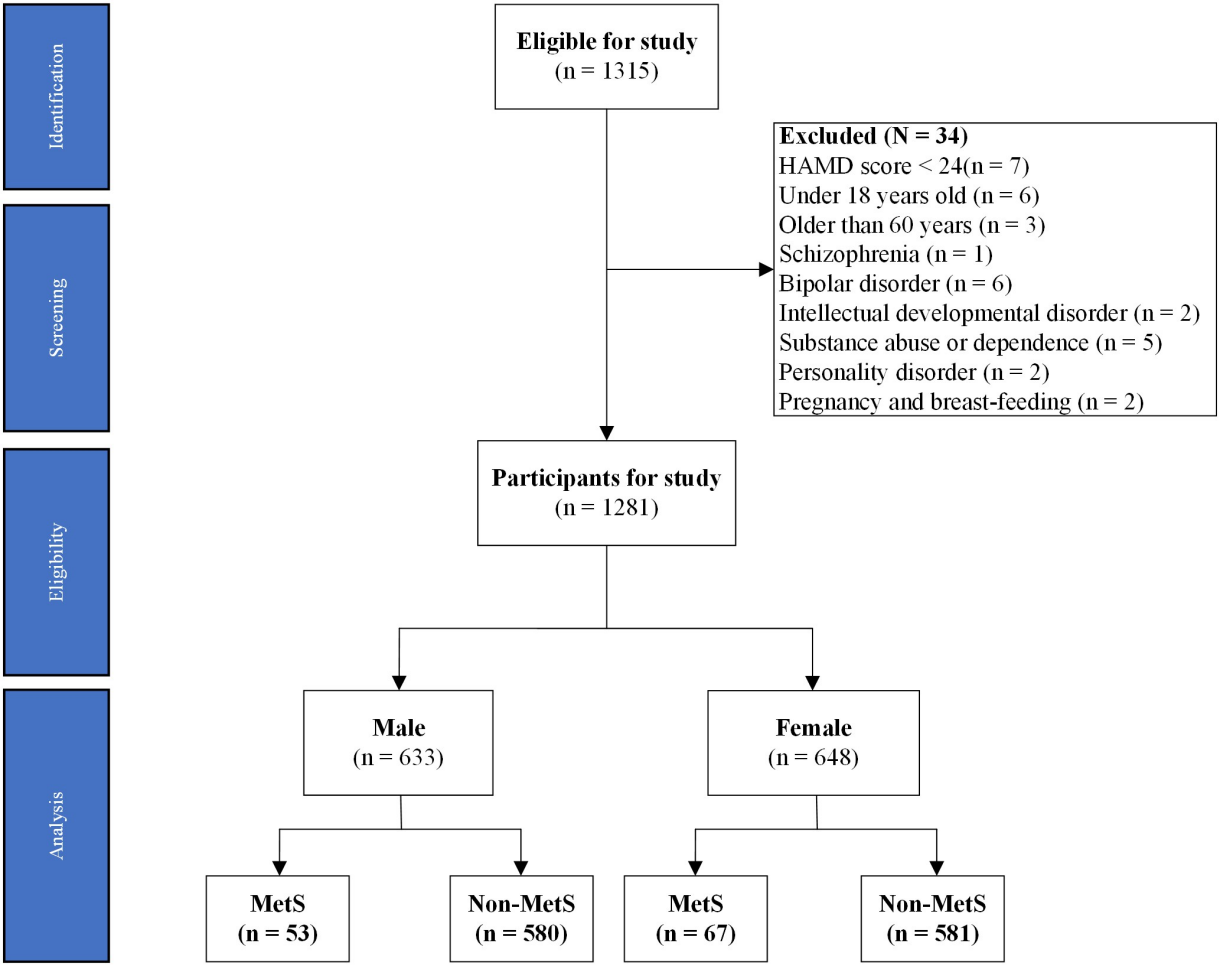
Flow diagram of participants in study. Note: HAMD: 17-item Hamilton Depression Rating Scale; MetS: metabolic syndrome.

The design of this study was a cross-sectional study. It aimed to analyze gender differences in the prevalence of MetS and related factors in first hospitalized MDD patients. Details of the data sources and the study flow are illustrated in Fig 1.

Upon admission, we collected relevant clinical data from patients diagnosed with MDD who met the inclusion criteria. The data collected included crucial factors such as age, gender, age of onset, illness duration, marital status, history of outpatient treatment, and the presence or absence of suicidal ideation. Moreover, we used standardized assessment tools to measure the severity of depressive symptoms with HAMD-17, anxiety symptoms with the Hamilton Anxiety Rating Scale (HAMA-14), psychotic symptoms using the Positive Symptom Subscale (PSS) from items P1 to P7 on the Positive and Negative Syndrome Scale (PANSS), and pre-treatment severity of the disease using the Clinical Global Impression Scale- Severity of Illness (CGI-SI).

On the second day after admission, venous blood is drawn from patients between 5:30 a.m. and 7:00 a.m. in a fasting state and sent to the hospital’s biochemistry laboratory by 9:00 a.m. for testing. The tests include lipid profiles, fasting blood glucose (FBG) levels, and thyroid function levels. The results are typically available from the biochemistry laboratory between 9:00 a.m. and 11:00 a.m. A detailed summary of these indicators is presented in Table 1.

**Table 1:** Demographic and general clinical data across different clinical subgroups.

Index	Total patients	Male	Female	*t*/*χ*^2^	*p—*value
	(n = 1281)	(n = 633)	(n = 648)		
Age—years	35.58±12.51	34.37±12.49	36.26±12.38	-2.26	0.024*
Onset age—years	33.99±12.48	32.82±12.37	34.74±12.31	-2.31	0.021*
Course of disease—months	10.77±4.50	10.96±4.55	10.76±4.34	0.69	0.488
MetS(Yes/No)	120/1161	53/580	67/581	2.00	0.158
Marital status (Married/Others)	867/414	420/213	457/191	2.58	0.108
Treatment history (Yes/No)	816/465	380/253	436/212	7.28	0.007*
Suicidal behavior (Yes/No)	170/1111	80/553	90/558	0.44	0.509
High school and below (Yes/No)	895/386	447/186	448/200	0.33	0.564
PSS	8.67±4.39	8.42±4.00	8.81±4.58	-1.37	0.171
CGI-SI	5.83±0.71	5.8±0.74	4.09±2.55	-0.71	0.478
HAMA	20.28±3.49	20.11±3.37	5.84±0.70	-1.10	0.274
HAMD	29.43±2.97	29.38±2.95	20.36±3.55	-0.45	0.656
FT_3_—pmol/L	4.9±0.69	4.94±0.70	16.81±2.96	1.37	0.171
FT_4_—pmol/L	16.78±3.04	16.72±3.18	0.01±0.35	-0.43	0.668
TSH—uIU/mL	3.98±2.47	3.76±2.31	4.88±0.69	-1.97	0.049*
MetS scores	0.00±0.35	-0.02±0.36	8.81±4.58	-1.42	0.675
MetS components					
WC—cm	80.16±8.39	81.84±8.88	79.03±7.99	5.03	< .001*
HDL-c—mmol/L	1.32±0.23	1.32±0.24	1.32±0.22	-0.05	0.875
TG—mmol/L	2.11±1	2.11±1.02	2.11±0.98	-0.04	0.96
FBG—mmol/L	5.26±0.63	5.26±0.63	5.26±0.63	0.69	0.971
SBP—mmHg	116.39±11.15	115.55±10.08	116.82±11.64	0.16	0.078
DBP—mmHg	74.62±6.83	74.47±6.94	74.7±6.77	-1.76	0.612
BMI—kg/m^2^	24.18±1.76	24.25±1.70	24.15±1.79	-1.06	0.421
LDL-c—mmol/L	2.76±0.78	2.63±0.73	2.68±0.75	-0.14	0.291
TC—mmol/L	4.80±0.90	4.78±0.84	4.79±0.97	-0.51	0.888

PSS: Positive symptom subscale; CGI-SI: Clinical Global Impression Scale—Severity of Illness; HAMA: Hamilton Anxiety Scale Score; HAMD: Hamilton Depression Scale score; FT_3_: Free triiodothyronine; FT_4_: Free tetraiodothyronine; TSH: Thyroid stimulating hormone; MetS: Metabolic syndrome; WC: waist circumference; HDL-c: high density lipoprotein cholesterol; TG: triglycerides; FBG: fasting blood glucose; SBP: systolic blood pressure; DBP: diastolic blood pressure; BMI: Body mass index; LDL-c: low density lipoprotein cholesterol; TC: total cholesterol.

*p<0.05

In China, the diagnostic criteria for MetS necessitate the presence of at least three out of five specific indicators [37]:

1. abdominal obesity, characterized by a waist circumference (WC) of ≥ 90 cm for men and ≥ 85 cm for women.
2. hyperglycemia, defined by FBG levels of ≥ 6.1 mmol/L or a diagnosis of diabetes mellitus.
3. hypertension, indicated by systolic blood pressure (SBP) of ≥ 130/85 mmHg, diastolic blood pressure (DBP) of ≥ 85 mmHg, or an established diagnosis of hypertension.
4. elevated triglycerides (TG) levels of ≥ 1.70 mmol/L.
5. low high-density lipoprotein cholesterol (HDL-c) levels below 1.04 mmol/L.

To comprehensively evaluate the severity of MetS within the target population, MetS was converted into a continuous variable. First, the mean arterial pressure (MAP) was calculated using the formula: MAP = 1/3×SBP + 2/3×DBP. Subsequently, MetS scores for patients with MDD of Han Chinese descent, across different genders, were computed based on the most recent research findings by *Shujuan Yang* et al. on Chinese adults [38].

Four psychiatrists, both holding at least the title of attending and affiliated with the study’s medical institution, conducted the psychological assessments. They were uniformly trained to ensure consistency in evaluation.

### 2.3. Data analysis

Categorical variables were reported as frequencies, whereas continuous variables with a normal distribution were presented as means with their corresponding standard deviations. Independent samples t-tests were utilized to compare continuous variables between groups, while the chi-square test was applied to compare proportions. To analyze gender differences in each clinical parameter, a 2 × 2 analysis of variance (ANOVA) was performed, considering gender (male and female) and diagnosis (with and without MetS) as factors. This approach allowed for the examination of the main effects of gender and diagnosis, as well as their interaction. Following this, separate ANOVAs were carried out within the male and female subgroups to compare clinical parameters between participants with and without MetS. Pearson correlation coefficients were calculated to investigate the relationships between MetS and other variables, excluding the components of MetS. Additionally, binary logistic regression analyses were performed to assess the association of MetS in both male and female participants. Finally, multivariate linear regression models were constructed to determine the correlates of MetS scores for men and women separately. All statistical tests were two-tailed, with a significance threshold set at < 0.05. Data analysis was conducted using SPSS 27.

## 3 Results

### 3.1 Demographic and general clinical data of enrolled patients

Table 1 presents the prevalence of MetS among male and female patients, which was 8.21% (52/633) and 10.34% (67/648), respectively, with no statistically significant difference observed between the two groups (F = 2.00, *p* = 0.158). Female patients demonstrated significantly higher age (t = -2.26, *p* = 0.024), onset age (t = -2.26, *p* = 0.024), and thyroid-stimulating hormone (TSH) levels (t = -1.97, *p* = 0.049) compared to male patients. In contrast, WC levels were significantly lower in female patients (t = 5.03, *p* < .001).

### 3.2. Gender differences in clinical characteristics associated with MetS

In Table 2, a 2×2 ANOVA was conducted to evaluate the interaction between MetS and gender. The analysis revealed significant gender differences in TSH and SBP levels, along with notable disparities in various clinical parameters, including psychological assessments (PSS scores, HAMD scores, HAMA scores, CGI-SI scores) and metabolic indicators (MetS scores, WC, FBG, TG, HDL-c, SBP, DBP, TC, low-density lipoprotein cholesterol [LDL-c]). Additionally, the interaction between gender and diagnosis significantly influenced SBP levels, with all *p*-values being less than 0.05.

**Table 2:** Comparison of demographic and clinical characteristics between patients with and without MetS, stratified by gender.

Parameters	MetS (n = 118)		Non-MetS (n = 595)		Gender	Diagnosis	Gender × diagnosis F (*p* -value)
	Male (n = 53)	Female (n = 67)	Male (n = 580)	Female (n = 581)			
					F (*p* -value)	F (*p* -value)	
Age—years	43.30±14.51	45.46±9.69	33.58±12.00	35.20±12.22	1.72(0.190)	48.11 (< .001*)	0.04 (0.848)
Onset age—years	41.93±14.06	44.31±9.66	32.02±11.90	33.64±12.11	1.97 (0.161)	55.06 (< .001*)	0.07(0.787)
Course of disease—years	11.50±5.62	9.90±5.05	10.92±4.46	10.86±4.25	2.48 (0.116)	0.12 (0.728)	2.13 (0.145)
PSS	12.37±7.13	13.06±7.16	8.07±3.41	8.31±3.90	0.88 (0.348)	82.37 (< .001*)	0.20 (0.657)
CGI-SI	6.15±0.82	6.18±0.82	5.77±0.72	5.80±0.68	0.11 (0.736)	20.00 (< .001*)	0.01 (0.976)
HAMA	22.81±3.71	22.85±4.34	19.87±3.24	20.08±3.33	0.09 (0.762)	50.00 (< .001*)	0.05 (0.830)
HAMD	31.52±3.93	31.6±3.39	29.19±2.78	29.22±2.82	0.03(0.872)	46.75 (< .001*)	0.01 (0.947)
FT_3_—pmol/L	4.79±0.61	4.91±0.70	4.95±0.71	4.89±0.70	1.14 (0.286)	0.59 (0.442)	0.15 (0.703)
FT_4_—pmol/L	15.85±2.88	16.84±2.95	16.80±3.20	16.81±2.97	1.91 (0.167)	1.59 (0.208)	1.86 (0.173)
TSH—uIU/mL	6.31±3.61	7.42±3.75	3.54±2.01	3.71±2.06	5.65 (0.018*)	45.28 (< .001*)	3.06 (0.081)
MetS scores	0.56±0.30	0.52±0.30	-0.07±0.32	-0.05±0.31	0.03 (0.864)	64.75 (< .001*)	0.80 (0.371)
MetS components							
** **WC—cm	86.41±9.72	83.21±7.81	81.44±8.70	78.55±7.87	9.73 (0.002*)	24.28 (< .001*)	0.02 (0.877)
** **HDL-c—mmol/L	1.19±0.28	1.22±0.22	1.33±0.23	1.33±0.22	0.20 (0.655)	22.63 (< .001*)	0.28 (0.597)
** **TG—mmol/L	2.86±1.28	2.61±0.99	2.05±0.97	2.06±0.97	1.03 (0.311)	34.07 (< .001*)	1.25 (0.264)
** **FBG—mmol/L	5.94±0.82	5.80±0.91	5.20±0.58	5.20±0.56	0.95 (0.329)	86.28 (< .001*)	0.94(0.333)
** **SBP—mmHg	127.3±9.48	134.21±9.18	114.52±9.47	114.81±10.13	9.41 (0.002*)	87.52 (< .001*)	7.93 (0.005*)
** **DBP—mmHg	84.7±9.41	84.33±8.28	73.57±5.90	73.59±5.61	0.06(0.809)	30.15 (< .001*)	0.08 (0.780)
BMI—kg/m^2^	24.58±1.5	24.27±1.91	24.22±1.72	24.14±1.78	0.83 (0.275)	1.40 (0.237)	0.28(0.600)
LDL-c—mmol/L	2.82±0.83	2.85±0.79	2.62±0.71	2.67±0.74	0.22 (0.638)	4.92 (0.027*)	0.01 (0.924)
TC—mmol/L	5.00±0.84	5.35±1.01	4.76±0.83	4.73±0.94	2.05(0.153)	15.73 (< .001*)	3.13(0.077)

PSS: Positive symptom subscale; CGI-SI: Clinical Global Impression Scale—Severity of Illness; HAMA: Hamilton Anxiety Scale Score; HAMD: Hamilton Depression Scale score; FT_3_: Free triiodothyronine; FT_4_: Free tetraiodothyronine; TSH: Thyroid stimulating hormone; MetS: Metabolic syndrome; WC: waist circumference; HDL-c: high density lipoprotein cholesterol; TG: triglycerides; FBG: fasting blood glucose; SBP: systolic blood pressure; DBP: diastolic blood pressure; BMI: Body mass index; LDL-c: low density lipoprotein cholesterol; TC: total cholesterol.

*p<0.05

### 3.3. Factors associated with MetS in male MDD patients

Table 3 presents the results of a correlation analysis among male patients, indicating that age (r = 0.21, *p* < .001), onset age (r = 0.21, *p* < .001), history of suicidal behavior (r = 0.21, *p* < .001), PSS scores (r = 0.21, *p* < .001), HAMD scores (r = 0.21, *p* < .001), HAMA scores (r = 0.21, *p* < .001), CGI-SI scores (r = 0.21, *p* < .001), and TSH levels (r = 0.21, *p* < .001) were positively correlated with MetS diagnosis. A binary logistic regression model (Backward: Wald) was subsequently used to identify independent variables (excluding MetS components) associated with MetS diagnosis, with age (B = 0.07, *p* < .001, OR = 1.07), HAMA scores (B = 0.13, *p* = 0.033, OR = 1.14), and TSH levels (B = 0.30, *p* < .001, OR = 1.35) emerging as significant risk factors (Table 4).Furthermore, a multiple linear regression model (Backward) was used to identify factors contributing to higher MetS scores, with onset age (B = 0.32, t = 6.65, *p* < .001, 95%CI = 0.01–0.02) and TSH levels (B = 0.34, t = 7.12, *p* < .001, 95%CI = 0.04–0.07) being significant contributors (Table 5).

**Table 3:** Correlation analysis between MetS and demographic/clinical variables in male and female patients.

Characteristic	Male (n = 633)		Female (n = 648)	
	r	*p*	r	*p*
Age—years	0.21	< .001*	0.25	< .001*
Onset age—years	0.22	< .001*	0.26	< .001*
Course of disease—years	0.04	0.523	-0.07	0.089
Married (No *vs*. Yes)	0.08	0.152	0.21	< .001*
Treatment history (No *vs*. Yes)	-0.01	0.930	-0.08	0.052
Suicidal behavior (No *vs*. Yes)	0.25	< .001*	0.27	< .001*
High school and above (No *vs*. Yes)	-0.02	0.678	-0.12	0.003*
PSS	0.29	< .001*	0.32	< .001*
CGI-SI	0.14	0.011*	0.17	< .001*
HAMA	0.24	< .001*	0.24	< .001*
HAMD	0.22	< .001*	0.24	< .001*
FT_3_—pmol/L	-0.01	0.820	-0.04	0.283
FT_4_—pmol/L	-0.08	0.137	0.01	0.922
TSH—uIU/mL	0.33	< .001*	0.44	< .001*
BMI—kg/m^2^	0.08	0.155	0.20	< .001*
LDL-c—mmol/L	0.08	0.161	0.08	0.053
TC—mmol/L	0.06	0.295	0.02	0.549

PSS: Positive symptom subscale; CGI-SI: Clinical Global Impression Scale—Severity of Illness; HAMA: Hamilton Anxiety Scale Score; HAMD: Hamilton Depression Scale score;FT_3_: Free triiodothyronine; FT_4_: Free tetraiodothyronine; TSH: Thyroid stimulating hormone; BMI: Body mass index; LDL-c: low density lipoprotein cholesterol; TC: total cholesterol.

*p<0.05

**Table 4:** Binary logistic regression analysis of MetS determinants in male and female patients.

	Coefficients	Std. error	Wald	*p* value	95% CI for EXP (B)		
	B				Exp(B)	Lower	Upper
Male							
Age—years	0.07	0.02	12.79	< .001*	1.07	1.03	1.11
HAMA	0.13	0.06	4.52	0.033*	1.14	1.01	1.28
TSH—uIU/mL	0.30	0.08	15.14	< .001*	1.35	1.16	1.57
Female							
Onset age	0.05	0.02	7.68	0.006*	1.05	1.01	1.08
Married (No *vs*. Yes)	2.79	1.08	6.66	0.010*	16.24	1.96	34.87
HAMD	0.10	0.06	2.98	0.084	1.10	0.99	1.23
TSH—uIU/mL	0.37	0.05	48.11	< .001*	1.45	1.31	1.61

HAMA: Hamilton Anxiety Scale Score; TSH: Thyroid stimulating hormone; HAMD: Hamilton Depression Scale score.

*p<0.05

**Table 5:** Factors influencing MetS scores in male and female patients: A Multiple linear regression model.

	Coefficients	Std. error	*t*	*p-*value	95% CI	
	B				Lower	Upper
Male						
Onset age—years	0.01	0.00	6.65	< .001*	0.01	0.02
TSH—uIU/mL	0.05	0.01	7.12	< .001*	0.04	0.07
Female						
Age—years	0.01	0.00	4.42	< .001*	0.00	0.01
Married (No *vs*. Yes)	0.12	0.03	3.65	< .001*	0.06	0.19
PSS	0.01	0.00	1.91	0.057	0.00	0.01
TSH—uIU/mL	0.05	0.01	9.53	< .001*	0.04	0.06

TSH: Thyroid stimulating hormone; PSS: Positive symptom subscale.

*p<0.05

### 3.4 Factors associated with MetS in female MDD patients

The correlation analysis revealed significant positive correlations with age (r = 0.25, *p* < .001), onset age (r = 0.26, *p* < .001), marital status (r = 0.21, *p* < .001), history of suicidal behavior (r = 0.27, *p* < .001), PSS scores (r = 0.32, *p* < .001), HAMD scores (r = 0.24, *p* < .001), HAMA scores (r = 0.24, *p* < .001), CGI-SI scores (r = 0.17, *p* < .001), TSH levels (r = 0.44, *p* < .001), and BMI levels (r = 0.20, *p* < .001). Conversely, higher education levels were negatively correlated with MetS (r = -0.12, *p* = 0.003) (Table 3). A binary logistic regression analysis (Backward: Wald) was then performed, including factors associated with MetS diagnosis (excluding MetS components) as independent variables. The analysis identified onset age (B = 0.05, *p* = 0.006, OR = 1.05), marital status (B = 2.79, *p* = 0.010, OR = 16.24), and TSH levels (B = 0.37, *p* < .001, OR = 1.45) as significant risk factors for MetS (Table 4). Additionally, multiple linear regression models (Backward) were constructed to determine the factors contributing to higher MetS scores, with age (B = 0.01, t = 4.42, *p* < .001, 95%CI = 0.00–0.01), marital status (B = 0.12, t = 3.65, *p* < .001, 95%CI = 0.06–0.19), and TSH levels (B = 0.05, t = 9.53, *p* < .001, 95%CI = 0.04–0.06) identified as significant contributors (Table 5).

## 4. Discussion

Our study revealed the following key findings: 1. The incidence of MetS in male and female MDD patients was 8.21% and 10.34%, respectively, with no significant gender difference. 2. Gender-specific differences were observed in the clinical factors contributing to MetS diagnosis. 3. Risk factors for MetS development also varied by gender: age and HAMA score were more significant for males, while onset age and marital status were more relevant for females. 4. Gender-specific variations were also noted in MetS severity, with onset age being more critical for males and age and marital status more influential for females.

There is currently no consensus regarding gender differences in the prevalence of MetS among individuals diagnosed with MDD, with geographic variability in study populations and subpopulations contributing to the inconsistent findings. For instance, a German study involving two independent MDD cohorts found that male patients had a significantly higher prevalence of MetS (30.2%-33.2%) compared to female patients (19.4%-22.1%) [39]. Another study focusing on refractory MDD showed no overall gender difference in MetS prevalence, but after adjusting for age, males over 40 years had a significantly higher prevalence than females (46.3% *vs*. 35.2%), while younger patients exhibited similar rates (27.3% *vs*. 31.8%) [40]. Conversely, research from Ethiopia, which included but did not exclusively focus on the MDD population, reported that males had an 88% lower prevalence of MetS compared to females [41]. In our study, the overall prevalence of MetS in the MDD population was notably lower than the aforementioned studies, and we found no significant gender difference.

Risk factors for MetS are often thought to be sex-differentiated, with the early stages of fetal sex differentiation laying the groundwork for this [42], and into adulthood, when estrogen becomes an additional cause of sex-differentiation [43, 44]. Our study identified depressive symptoms only as a risk factor for MetS in female patients. In a large representative British birth cohort, the role of adolescent mood problems in significantly predicting adult MeS was confirmed only in female patients [45]. Another large sample meta-analysis reported that baseline depressive symptoms were more strongly associated with predicting MetS in female patients and not significant in predicting male patients [46]. Interestingly, we also found that married status was a risk factor for co-morbid MetS only in female patients, but not in males. A U.S. study found that marital quality was indirectly associated with MetS through depressive symptoms, but there were no gender differences in this association [47]. This is something that we have not found similar reports on, and whether this is related to specific cultural characteristics in China needs to be determined through an in-depth rigorously designed study.

Beyond identifying the relationship between various clinical parameters and the dichotomized MetS, we also quantified MetS to assess the dose-response relationship with clinical parameters. However, similar studies in MDD populations are scarce. Some researchers have reported an increase in the number of MetS risk factors among women experiencing more severe depressive symptoms [48]. Additionally, two large cohort studies found that patients with more severe depression tended to have a greater number of MetS components [49, 50]. While these studies partially support our findings, they do not specifically address gender differences. *Kuan Zeng* et al. explored gender differences in MetS severity among patients with schizophrenia, not MDD, and although the specific risk factors identified differed from our study, they similarly concluded that female patients exhibited more numerous and widespread risk factors for MetS severity [51]. This aligns somewhat with our findings. Overall, our study found a gender difference in MetS severity among first hospitalized MDD patients.

Our study has certain limitations that should be acknowledged. First, the cross-sectional nature of the research limits our ability to draw causal inferences between the variables examined. Second, Univariate analysis found that the mean age was higher in the female group, which may have contributed to the high rate of MetS reporting in this group. Third, the inclusion of patients with varying treatment histories introduced potential confounding variables that could affect the results. Future research should address these limitations through well-designed prospective studies to provide more robust conclusions.

## 5. Conclusion

Our study did not identify a significant gender disparity in the prevalence of MetS among first hospitalized MDD patients. Nevertheless, substantial gender differences were detected in both the presence and severity of MetS, with distinct risk factors emerging for male and female patients. For males, the risk of developing MetS is higher in those with older age and higher anxiety levels, while the severity of MetS increases in those with an older age of onset. In contrast, for females, there is a greater need to focus on the development of MetS in those with an older age of onset and married status. Additionally, the severity of MetS in females is more pronounced in those who are older and married, highlighting the importance of addressing these specific factors in female patients when managing MetS in the MDD population.
